# Editorial: The Temporal Dynamics of Cognitive Processing

**DOI:** 10.3389/fpsyg.2016.00930

**Published:** 2016-06-21

**Authors:** Timothy M. Ellmore, Peter F. Dominey, John F. Magnotti

**Affiliations:** ^1^Psychology, The City College of New YorkNew York, NY, USA; ^2^Centre National de la Recherche Scientifique, Inserm U846Lyon, France; ^3^Neurosurgery, Baylor College of MedicineHouston, TX, USA

**Keywords:** memory, attention, cognitive control, aging, social decision making

From our ability to attend to many stimuli occurring in rapid succession to the transformation of memories during a night of sleep, cognition occurs over widely varying time scales spanning milliseconds to days and beyond. Cognitive processing is often influenced by several behavioral variables as well as non-linear interactions between multiple neural systems. This frequently produces unpredictable patterns of behavior and makes understanding the underlying temporal factors influencing cognition a fruitful area of hypothesis development and scientific inquiry. Across two reviews, a perspective, and 12 original research articles covering the domains of learning, memory, attention, cognitive control, and social decision making this research topic sheds new light on the temporal dynamics of cognitive processing.

Highly relevant to a fundamental understanding of behavior is how prediction errors (PEs), the differences between predicted and actual effects, modulate learning. Bayesian models of associative learning include PEs as a critical variable, while other models posit PEs as key to uncertainty-related learning as well as cognitive control. Limongi et al. hypothesized that temporal PEs might disrupt behavior-change performance under uncertainty. Subjects made temporal predictions while observing a moving ball strike a stationary ball, which deflected at a variable temporal gap, and in some trials, a change signaled them to alter their behaviors. Performance accuracy fell as a function of both temporal PE and the delay. The authors interpret from a free energy perspective the participants' inaccurate prepotent behavior as compensation for imprecise perceptual inference.

Stress is a critical variable influencing learning both positively and negatively, especially when a stressful episode comes immediately before or right after learning. Cadle and Zoladz present the idea and review data suggesting that learning is enhanced in some instances because the learned information becomes a part of a “stress context” and is subsequently tagged by the emotional memory being formed. However, when the stressful episode is separated in time from learning, the memory is impaired because the learning is experienced outside the context of the stress episode.

Daniel et al. report on how pigeons use time to alternate between two opposing rules, matching and non-matching, using a mid-session reversal task. In the first half of a session, pigeons performed a match-to-sample task, which then switched to non-match-to-sample for the second half. Pigeons that had previously learned either a matching or non-matching abstract concept were trained in this mid-session reversal task. After training, novel stimuli were inserted into either the early (matching-to-sample) or late (non-match-to-sample) part of the session. Birds responded to these novel trials according to the previously-learned abstract concept, independent of the time of the session, while still responding to trained stimuli according to the current rule (matching vs. non-matching). Their study shows that pigeons are an excellent model for understanding the comparative processes that underlie the temporal dynamics of discrimination learning and concept formation.

Escobar et al. investigate the associative structure of long-delay conditioning using a rat model. In long-delay conditioning, a stimulus of long duration elicits a conditioned response primarily in its latter stages, rather than throughout the stimulus. Of theoretical importance is whether the initial segment of a long-delay conditioned stimulus actually acquires inhibitory strength. Using an appetitive conditioning procedure, they tested for conditioned inhibition and found that the initial segment of a long delay CS appears to share more characteristics with a latent inhibitor than a conditioned inhibitor. The findings are consistent with componential theories of conditioning. They conclude that stimuli must not be viewed as unitary events, but instead as a sequence of temporally linked events, each link carrying different types of information about the conditions that precede and follow them.

Working memory includes the ability to hold information in mind that is no longer physically present, and to manipulate it to achieve goals. Kaiser provides a much needed review of electro- and magneto-encephalographic studies of auditory working memory. Studies of spatial and non-spatial auditory working memory have found differential roles for ventral and dorsal stream regions, while event-related potentials reveal sustained memory load-dependent deflections during retention with activation peaks during delay periods related to task performance. The findings highlight that auditory working memory relies on the dynamic interplay between frontal executive and sensory representation systems.

Which working memory strategies best offset age-related declines in fluid cognition? Basak and O'Connell tackle this question by studying older adults who were randomly assigned to one of two types of working memory training. One group was trained on a predictable memory updating task while another was trained on a novel, unpredictable memory updating task. Compared to predictable training, unpredictable memory updating requires greater demands on cognitive control. The results obtained showed significantly enhanced performance on episodic memory and faster learning of a new working memory task after the unpredictable training. Unpredictable memory updating training may offer a better way to improve cognitive abilities in aging adults.

Knowledge of one's own memory abilities involve processes that are built over time. Chua and Solinger focus on how metamemory processes depend on different prospective and retrospective factors across the learning and memory timescale. By analyzing feeling-of-knowing judgments about target retrievability and retrospective confidence judgments about retrieved targets, they conclude that metamemory judgments should not be thought of as discrete experiences in time, but instead as an evolving awareness that dynamically incorporates previous judgments with new information.

Consolidation, the stabilization and strengthening of memories, also evolves over time often during periods of “offline” processing when the participant isn't engaged in similar information processing. The behavioral factors that influence how offline processing results in the temporal stabilization of memories are not well understood. Ellmore et al. tested different groups of subjects after each was exposed to novel visual stimuli. One group remained in the lab and engaged in an attention demanding task. Another group remained in the lab and rested quietly. Yet another group left the lab and returned later for testing. The different manipulations during the offline processing period affected long-term recognition of the visual stimuli a day later. Results indicated that remaining in the same context and resting quietly with minimal engagement of attention results in the best ability to distinguish old from novel visual stimuli.

How does attention affect processing of time? Using a temporal variant of the mismatch negativity (MMN) paradigm, Campbell and Davalos studied temporal processing by introducing rare, deviant interstimulus intervals amid a series of standard intervals and recorded EEG. They manipulated subjects' level of attention to the intervals and the difficulty of the task (size of the deviant interval). They found an interaction between task difficulty and attention—the largest neurophysiological responses came from the conditions with high attention and the largest deviants. Their results reiterate the importance of attention in temporal and memory processing generally and may provide a way to assess differences in temporal processing between individuals and groups.

Limited attentional capacities are reliably demonstrated with the attentional blink, a task in which humans miss a second target when it should be detected within 600 ms of an initial target. Dynamic attention theory posits that attention cycles in an oscillatory manner with regular pulses evoking expectations regarding the point of the next occurrence of a tone in a rhythm. This allows for more attentional resources to be provided. New findings reported by Bermeitinger and Frings suggest that oscillatory cycling attention does not affect temporal selection as tapped in an AB paradigm, indicating the need for future research to test whether regular and irregular rhythms and long/stronger entrainment phases differ in their influence on the AB effect.

Multitasking is ubiquitous in today's world, and a more thorough understanding of how multiple task demands presented closely in time affect performance is highly relevant. Completion of multiple tasks often affects performance negatively, as the prioritized task can be detrimentally affected by additional tasks. Scherbaum et al. conduct a dynamic investigation of two tasks, Task 1 and Task 2, in a dual-task setting. An additional Task 2 interfered with Task 1, with the influence dependent on the temporal proximity of task stimuli. The influence onto Task 1 performance was continuous and irrespective of any critical window of influence. Furthermore, there was modulation of crosstalk by previous interference, which indicates flexible adaptation of task-shielding. Finally, the execution of Task 1 was influenced by previously executed responses of both tasks and the influences showed different temporal patterns, indicating a sustained reactivation of the previous response of Task 1.

Multitasking abilities also depend critically on cognitive flexibility, a component of executive functions allowing us to behave in dynamic environments. Dshemuchadse et al. distinguish between shifting flexibility and spreading flexibility. Using a homonym relatedness judgment task combined with mouse tracking, they demonstrate that these two types of cognitive flexibility follow independent temporal patterns in their influence on participants' mouse movements during the relatedness judgments. The results demonstrate the need for future studies to consider shifting and spreading flexibility independently when studying moderators of cognitive flexibility.

Noticing how the environment changes over time is critical for survival. A large body of literature suggests that when these changes occur during a blank temporal interval, they can go unnoticed, a phenomenon called change blindness. Herbranson reports that pigeons also experience such change blindness, suggesting commonalities across species in how visual scenes are processed. Like humans, pigeons show sensitivity to the presence of a blank interval between the original and unchanged displays and performance increases as the number of repetitions increases. Pigeons also appear to engage in a serial search for changes, as additional time is required to search additional locations.

In social decision making, the ability to integrate moral intention information with the outcome of an action plays a crucial role in mature social judgment. This integration process occurs across time, but is likely to be quick. Gan et al. show evidence for fast moral intuition reactions and subsequent integration processing in the right temporo-parietal area. Participants made moral judgments for agents who produced either negative/neutral outcomes with harmful/neutral intentions or positive/neutral outcomes with helpful/neutral intentions. Neural differences measured with EEG between attempted and successful actions over prefrontal and bilateral temporo-parietal regions were found in both harmful and helpful moral judgment with a neural integration time course from right temporo-parietal area to left temporo-parietal area, then to prefrontal and right temporo-parietal area again.

The aim of this research topic was to understand basic temporal aspects of cognitive processing more thoroughly. To understand the temporal structure of the underlying neural processes, however, it is clear that further methodological development will be needed. Kadipasaoglu et al. report on the development of robust techniques for group analysis of intracranial EEG data, a powerful new method to investigate the architecture of interregional dynamics of distributed cortical networks. An illustration of this work is featured as the cover of the research topic. The combination of structured cognitive tasks, the high spatial and temporal resolution of intracranial EEG, and powerful within- and between-subjects statistical inference will no doubt help contribute to a more detailed understanding of how cognition plays out in time.

## Author contributions

TE, PD, and JM have made substantial, direct and intellectual contributions to the work, and approved it for publication.

## Funding

TE is supported by the National Institute of General Medical Sciences of the National Institutes of Health under Award Number SC2GM109346. The content is solely the responsibility of the authors and does not necessarily represent the official views of the National Institutes of Health.

### Conflict of interest statement

The authors declare that the research was conducted in the absence of any commercial or financial relationships that could be construed as a potential conflict of interest.

